# The *Drosophila* speciation factor HMR localizes to genomic insulator sites

**DOI:** 10.1371/journal.pone.0171798

**Published:** 2017-02-16

**Authors:** Thomas Andreas Gerland, Bo Sun, Pawel Smialowski, Andrea Lukacs, Andreas Walter Thomae, Axel Imhof

**Affiliations:** 1 Biomedical Center, Histone Modifications Group, Department of Molecular Biology, Ludwig-Maximilians-Universität München, Planegg-Martinsried, Germany; 2 Center for Integrated Protein Science Munich (CIPSM), Ludwig-Maximilians-Universität München, Munich, Germany; 3 Biomedical Center, Core Facility Computational Biology, Ludwig-Maximilians-Universität München, Planegg-Martinsried, Germany; 4 Biomedical Center, Core Facility Bioimaging, Ludwig-Maximilians-Universität München, Planegg-Martinsried, Germany; Oxford Brookes University, UNITED KINGDOM

## Abstract

Hybrid incompatibility between *Drosophila melanogaster* and *D*. *simulans* is caused by a lethal interaction of the proteins encoded by the *Hmr* and *Lhr* genes. In *D*. *melanogaster* the loss of HMR results in mitotic defects, an increase in transcription of transposable elements and a deregulation of heterochromatic genes. To better understand the molecular mechanisms that mediate HMR’s function, we measured genome-wide localization of HMR in *D*. *melanogaster* tissue culture cells by chromatin immunoprecipitation. Interestingly, we find HMR localizing to genomic insulator sites that can be classified into two groups. One group belongs to *gypsy* insulators and another one borders HP1a bound regions at active genes. The transcription of the latter group genes is strongly affected in larvae and ovaries of *Hmr* mutant flies. Our data suggest a novel link between HMR and insulator proteins, a finding that implicates a potential role for genome organization in the formation of species.

## Introduction

Biodiversity is the result of the emergence and the extinction of species. New species form by pre- and post-zygotic isolation mediated by genetic incompatibility [1]. One of the best characterized examples of hybrid incompatibility is the gene pair *Hybrid male rescue* (*Hmr*) and *Lethal hybrid rescue* (*Lhr*). *Hmr* and *Lhr* cause hybrid incompatibility between the closely related fly species *Drosophila melanogaster* and *D*. *simulans*. *Hmr* diverged in both *Drosophila* sibling species under positive selection [2]. HMR and LHR from both species interact physically and localize predominantly to centromeric regions [3]. A reduction of HMR expression results in a misregulation of transposable elements, satellite DNAs and heterochromatic genes [3–5]. The major difference between HMR and LHR in *D*. *melanogaster* and *D*. *simulans* is their substantial difference in protein amounts [3,6], which has been proposed to result in a lethal gain of function in male hybrids [3]. High levels of HMR and LHR in hybrids and overexpression of these proteins in pure species lead to an increased number of binding sites of the complex [3]. Such spreading phenomena based on protein amount have been observed for several chromatin-associated complexes such as the dosage compensation complex [7,8], the polycomb complex [9] or components of pericentromeric heterochromatin [10,11]. In most cases, the precise mechanisms for targeting and spreading are not fully understood. Interestingly, several of the components involved in these processes show signs of adaptive evolution and differ substantially even in very closely related organisms [12–14]. This observation has spurred a model of a dynamic genome that drives the adaptive evolution of chromatin-associated factors [15].

Eukaryotic genomes of closely related species differ mostly in the amount and sequence of repetitive DNA [16–18]. This DNA is often derived from transposable elements, which are highly mutagenic and are therefore under tight transcriptional control by the cellular machinery. During evolution transposons or transposon-derived sequences occasionally adopted structural or novel *cis*-regulatory functions, thereby contributing to the evolution of new, species-specific, phenotypic traits [19–21]. Genomic insulators are a particular class of such novel, fast evolving, *cis*-regulatory elements that show signs of transposon ancestry [22,23]. A strong expansion of these elements is observed in arthropods, which also experienced a successive gain in the number of insulator binding proteins during evolution [24]. In fact, the *Drosophila* genome harbours a large variety of insulator proteins such as CTCF, BEAF-32, Su(Hw), Mod(mdg4) and CP190, which all affect nuclear architecture [25]. Different *Drosophila* species underwent multiple genomic rearrangements and transposon invasions [26,27], which presumably resulted in an adaptive response of regulatory DNA binding factors to maintain spatial and temporal gene expression. For example, binding sites for the insulator proteins BEAF-32 and CTCF show a high degree of variability when compared among very closely related species [26,27]. The gain of new insulator sites is associated with chromosome rearrangements, new born genes and species-specific transcription regulation [19,23]. Similar to insulator proteins, which tend to cluster in specific nuclear regions [28], the speciation factor HMR clusters at centromeres or pericentromeric regions in diploid cells [3,6] but is also detected at distinct euchromatic regions along the chromosome arms in polytene chromosomes [3]. A unifying feature for many of these sites is their close proximity to binding sits of the Heterochromatin Protein 1 (HP1a), a HMR interactor and a well-characterized heterochromatic mark.

Various studies describe HMR’s localization to heterochromatin, but the molecular details on HMR’s binding sites and its recruitment to these sites are not well understood. To get new insights into HMR’s association to chromatin, we measured HMR’s genome-wide localization by chromatin immunoprecipitation (ChIP) in the *D*. *melanogaster* embryonic S2 cell line. We demonstrate an extensive colocalization of HMR with a subset of insulator sites across the genome. HMR’s binding to genomic *gypsy* insulators, which constitute the major group of its binding sites, is dependent on the residing insulator protein complex. In a second group, HMR borders heterochromatin together with the insulator protein BEAF-32. In agreement with previous low-resolution techniques in cell lines and fly tissue [3], these binding sites are enriched at pericentromeric regions, the cytological region 31 on the 2nd chromosome and the entire 4th chromosome. At most of these sites, HMR associates to the promoters of actively transcribed genes. Interestingly, these genes code for transcripts that have been reported to be downregulated in *Hmr* mutant larvae and ovaries. Altogether, our data provide evidence for a functional link between HMR and insulator proteins, which potentially results in hybrid incompatibilities due to the adaptive evolution of these genome-organizing complexes.

## Materials and methods

### Cell culture and RNAi

*D*. *melanogaster* S2-DRSC cells were obtained from the DGRC and grown at 26°C in Schneider's *Drosophila* medium (Invitrogen) supplemented with 10% fetal calf serum and antibiotics (100 units/mL penicillin and 100 μg/mL streptomycin).

For RNAi experiments cells were incubated in serum-free medium containing 10 mg/mL dsRNA. After 1 hr of incubation, the serum-containing medium was supplied. Samples were taken after 7 days. The dsRNA was prepared using the MEGAScript T7 Transcription Kit (Thermo Fisher Scientific) following the manufacturers instructions with primers listed in S1 Table.

### Chromatin immunoprecipitation, Real-Time PCR and sequencing

For chromatin immunoprecipitation (ChIP) cells were crosslinked with 1% formaldehyde for 5 min at room temperature. Upon cell lysis, protease inhibitors and proteasome inhibitor MG-132 (Enzo Life Sciences) were applied. The chromatin was isolated and sheared with adaptive focused acoustics (Covaris) to an average size of 200 base pair (bp). For each ChIP reaction, chromatin isolated from 1–2 x 10^6^ cells was incubated with following antibodies precoupled to Protein A/G Sepharose: rat anti-HMR 2C10 (RRID: AB2569849) [3] with rabbit IgG anti-rat IgG (RRID: AB2339804), mouse anti-HP1a C1A9 (RRID: AB528276) [29], rabbit anti-H3 (RRID: AB302613), rabbit anti-H3K9me3 (RRID:AB2532132) and mouse anti-FLAG (RRID: AB262044). Real-Time PCR was performed with Fast SYBR Green master mix (Applied Biosystems) using a LightCycler 480 II (Roche). For deep sequencing, all libraries were prepared using MicroPlex (Diagenode) or NEBNext (NEB) Library Preparation kit and single-end, 50 bp sequenced with the Illumina HiSeq2000. An overview of all ChIP-Seq samples used and the number of uniquely aligned sequence reads is provided as S2 Table. A list of HMR peaks used for further analyses is provided as S3 Table. All sequencing data are publicly available as described below.

### Data analysis

The raw reads were aligned to the *D*. *melanogaster* genome assembly (dm3) using Bowtie 2.2.6 with unique mapping criteria and exclusion of chromosome Uextra [30]. The raw read quality was accessed using FASTQC 11.5 [31] and read filtering was performed using FastX 0.0.13 [32]. Sequencing tracks were visualized using IGB [33] and IGV [34] genome viewers. Peak calling, motif search and peak annotation were performed using HOMER 4.8 with peak size of 200 bp [35] and ChIPseeks implementation of HOMER [36]. For downstream analysis, peaks identified in two out of three biological replicates were taken. Downstream analysis steps were performed using Python and R and parts of data preprocessing was done using ChipPeakAnno [37]. For repeat analysis, reads from ChIP-Seq experiments were mapped to RepBase version 19.10 [38] using Bowtie [30]. Only unique reads were kept for analysis. For each repetitive element, the log2 fold change was calculated. Following genome-wide binding data sets derived from S2 cells (unless stated otherwise) were used: CP190, Su(Hw), CTCF and mod(mdg4) from GEO GSE41354 [39], BEAF-32 from GEO GSE32815 [40]. RNA expression data for untreated S2 cells was taken from GEO GSE46020. For *D*. *melanogaster* larvae and ovaries, RNA-Seq data were taken from NCBI BioProject PRJNA236022 [4] and analyses were performed with cuffdiff 2 [41]. An extended description of the bioinformatics tools and methods used is provided in S1 Methods.

### Western blot analysis

Samples were boiled in loading buffer, separated on SDS-PAGE gels (Serva), processed for western blot using standard protocols and detected using rat anti-HMR 2C10 (1:20) (RRID: AB2569849), rabbit anti-CP190 (RRID: AB2615894) [42], rabbit anti-H3K9me3 (1:2000) (RRID:AB2532132) and mouse anti-Tubulin (1:800) (RRID: AB2241150) antibodies. Secondary antibodies included sheep anti-mouse (1:5000) (RRID: AB772210), goat anti-rat (1:5000) (RRID: AB772207), donkey anti-rabbit (1:5000) (RRID: AB772206) coupled to horseradish peroxidase.

### Data access

ChIP-Seq data from this study are publicly available at NCBI GEO (GSE86106).

## Results

### Genome-wide binding map of HMR in *D*. *melanogaster*

**Fig 1:**
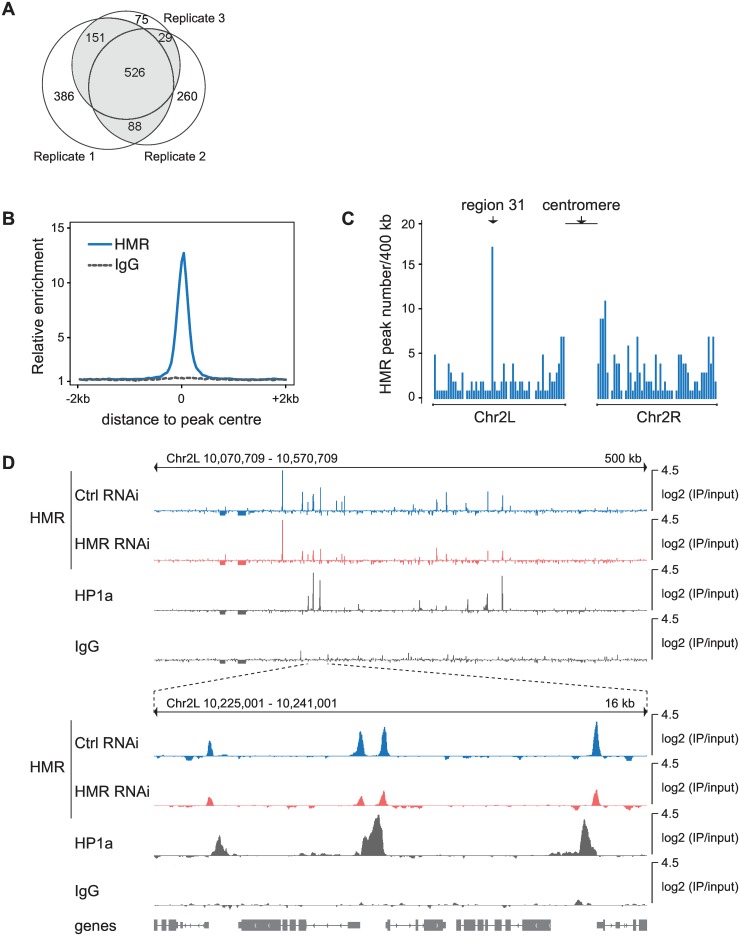
Identification of HMR binding sites in *D*. *melanogaster* S2 cells. **(A)** Venn diagram of HMR peaks showing the number of peaks identified in three independent biological replicates. Peaks identified in at least two out of three replicates were used for further analysis and are highlighted in *grey*. **(B)** Composite analysis of HMR and control IgG (anti-FLAG) ChIP signals at genomic HMR peak positions. **(C)** Histogram of HMR peak density across the left arm (2L) and right arm (2R) of the 2nd chromosome. The cytological region 31 and centromere-proximal regions are indicated **(D)** Genome browser view of HMR, HP1a and control IgG (anti-FLAG) ChIP signals at region 31. HMR ChIP signals obtained upon knock-down using control RNAi and HMR RNAi are shown with the same amplitude.

Immunohistological studies revealed a binding of HMR to centromeric or pericentromeric regions in diploid cells and to several euchromatic and telomeric regions in polytene chromosomes [3,6]. However, detailed information on HMR’s binding to chromatin was so far lacking. To better understand the molecular mechanisms that govern HMR’s binding within the genome, we mapped the genomic binding sites of HMR in cultured *D*. *melanogaster* S2 cells. We used a highly specific monoclonal antibody against HMR [3] to purify associated chromatin followed by next generation sequencing (ChIP-Seq) and derived a set of 794 HMR binding sites, which were present in at least two out of three biological replicates (Fig 1A). A composite plot of all HMR binding sites found in the genome revealed a sharp peak of HMR binding with a width of approximately 200 nucleotides, which is reminiscent of sequence specific transcription factors (Fig 1B). To validate the identified HMR binding sites, we applied multiple strategies. First, we measured enrichment of the HMR binding sites in ChIP experiment using an anti-FLAG antibody, an epitope that is not expressed in wild type cells (Fig 1B and S1A Fig). Second, we performed RNAi knock-down experiments to reduce HMR protein level and compared the enrichment of HMR between HMR RNAi treated cells and Control (Ctrl) RNAi treated cells. Although we observe an overall reduction of HMR binding at most HMR peaks (S1B Fig), we rarely see a complete loss of binding despite the high efficiency of the HMR knock-down. This apparent discrepancy suggests that chromatin-bound HMR is rather resistant towards a RNAi-mediated removal. The existence of such RNAi-resistant binding sites in ChIP experiments has been observed before and was attributed to high-affinity binding sites [43] or an incomplete removal of the chromatin-bound factors. Third, we used the CRISPR/*cas9* system to edit the HMR locus in S2 cells such that the cell line exclusively expresses an HMR allele, which carries a double FLAG-tag at the C-terminus. ChIP-qPCR using HMR and FLAG antibody in wild type and HMR-Flag_2_ expressing cells showed specific and reproducible enrichment of HMR at selected HMR binding sites (S1C Fig).

We find HMR binding sites on all chromosomes and distributed along the whole chromosome arms with a marked increase in peak density at pericentromeric regions and at the 4th chromosome where we also observe a higher density of binding sites for HP1a, a known interaction partner of HMR [3,4] (Fig 1C and S1D Fig). Unfortunately, the centromeric regions are not present in the current *Drosophila* genome assembly, preventing read mapping and analysis in this area of the genome. However, the increased number of peaks at pericentromeric regions (S1D Fig) is consistent with the strong centromeric HMR signal we previously observed when staining S2 cells with an anti-HMR antibody [3]. Besides the pericentromeric region, we also observe a strong clustering of HMR peaks at the cytological region 31 on the left arm of the second chromosome where we also see HMR binding in polytene chromosomes [3]. Interestingly, HMR binding sites within these regions do not completely overlap with HP1a bound regions but rather localize at their edges (Fig 1D).

### HMR binding sites largely overlap with genomic insulator sites

**Fig 2:**
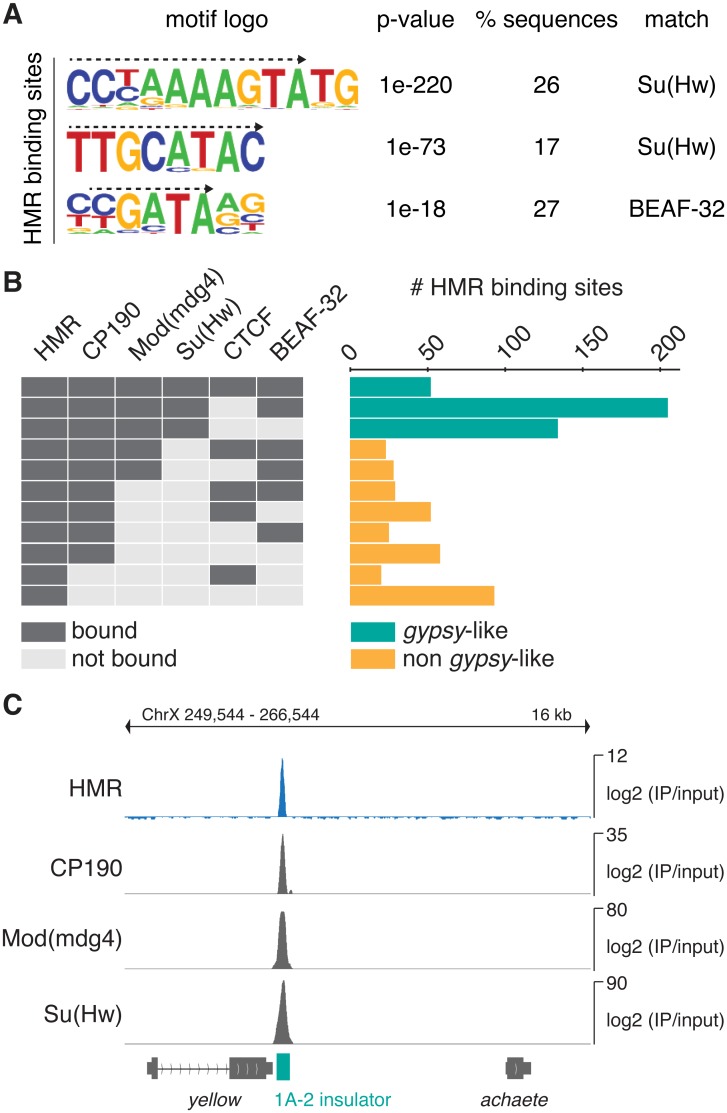
HMR localizes to genomic insulators and the *gypsy* transposon. **(A)** Sequence motifs identified within HMR peak regions. The corresponding motif logo, p-value of enrichment, percentage of regions with this motif and putative binding factors are indicated. Dashed arrows mark the sequence that matches the published binding sites of Su(Hw) and BEAF-32 (see also S2A Fig) **(B)** Peak overlap of HMR with peaks of the insulator proteins CP190, Mod(mdg4), Su(Hw), CTCF [39] and BEAF-32 [40]. The number of HMR peaks is indicated depending on their colocalization with known boundary factors. Groups with less than 11 members are not displayed. Su(Hw)-containing *gypsy*-like groups are depicted in *green*, non *gypsy*-like groups in *orange*. Combinations that contain less than ten HMR peaks are not shown. **(C)** Genome browser view of the Su(Hw) binding region 1A-2. ChIP signals of HMR and known *gypsy* binding factors are shown. The 1A-2 insulator is highlighted in *green*.

We next asked whether HMR binding sites are enriched for specific DNA sequence motifs. A motif analysis of HMR-bound regions revealed three DNA sequence motifs that were significantly enriched and present in up to 26% of all HMR peaks (Fig 2A). These motifs are highly related to the recognition motifs of the insulator DNA binding proteins Su(Hw) and BEAF-32 (S2A Fig), both containing a zinc-finger DNA-binding domain [44,45], suggesting that HMR binds to insulator regions. Indeed, we observe a substantial overlap of our HMR binding profiles with the published ones of known insulator proteins such as CP190, Mod(mdg4), Su(Hw), CTCF and BEAF32 [39,43] (Fig 2B and S2B Fig). Insulator binding sites can be subclassified depending on their composition of known insulator proteins [43]. One of the best characterized family of insulators are derived from the *gypsy* retrotransposon and are strongly bound by Su(Hw), Mod(mdg4) and CP190 [46–48]. Consistent with the strong enrichment of Su(Hw)-recognition motifs in the binding sites of HMR, we find about half of all HMR sites belonging to this *gypsy*-like family of insulators (Fig 2B). However, only 7% of all Su(Hw) binding sites and 11% of *gypsy*-like elements classified as bound by Su(Hw), Mod(mdg4) and CP190 are also bound by HMR.

**Fig 3:**
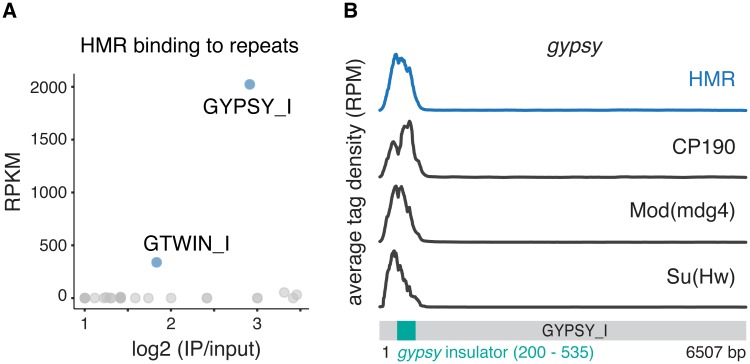
HMR localisation to repetitive elements. **(A)** HMR ChIP tag enrichment at repetitive DNA elements. To identify enriched sequences the enrichment (log2-fold) over input is plotted against the RPKM of an individual repeat sequence from RepBase. Repeats with less than 2-fold enrichment are not displayed. **(B)** ChIP tag density of HMR and the *gypsy*-insulator proteins CP190, Mod(mdg4), Su(Hw) [39] across the repetitive *gypsy* retrotransposon sequence. The *gypsy* insulator sequence at the 5' end is highlighted in *green*.

Given the extensive colocalization of HMR with non-repetitive *gypsy*-like insulators (Fig 2C) and the effect of a *Hmr* mutations on the expression of retrotransposons [3,4], we wondered whether HMR is also enriched at repetitive DNA. We therefore mapped sequences obtained from our ChIP-Seq experiments using anti-HMR and anti-HP1a antibodies as well as published binding profiles for Su(Hw), Mod(mdg4)2.2 and CP190 [39] against the RepBase repeat database [38]. In agreement with previous studies we observe a strong enrichment of HP1a at the centromeric heterochromatin-associated Dodeca satellite (DMSAT6) [49] and the transposable elements Rt1a and Rt1b (DMRT1A, DMRT1B) [50] (S2C Fig). In contrast to HP1a, the only repetitive elements that show a substantial enrichment for HMR are the retrotransposons *gypsy*, and *gtwin* (Fig 3A). At these elements HMR binds together with Su(Hw), Mod(mdg4) and CP190 to the 5’ insulator region (Fig 3B and S2D Fig).

**Fig 4:**
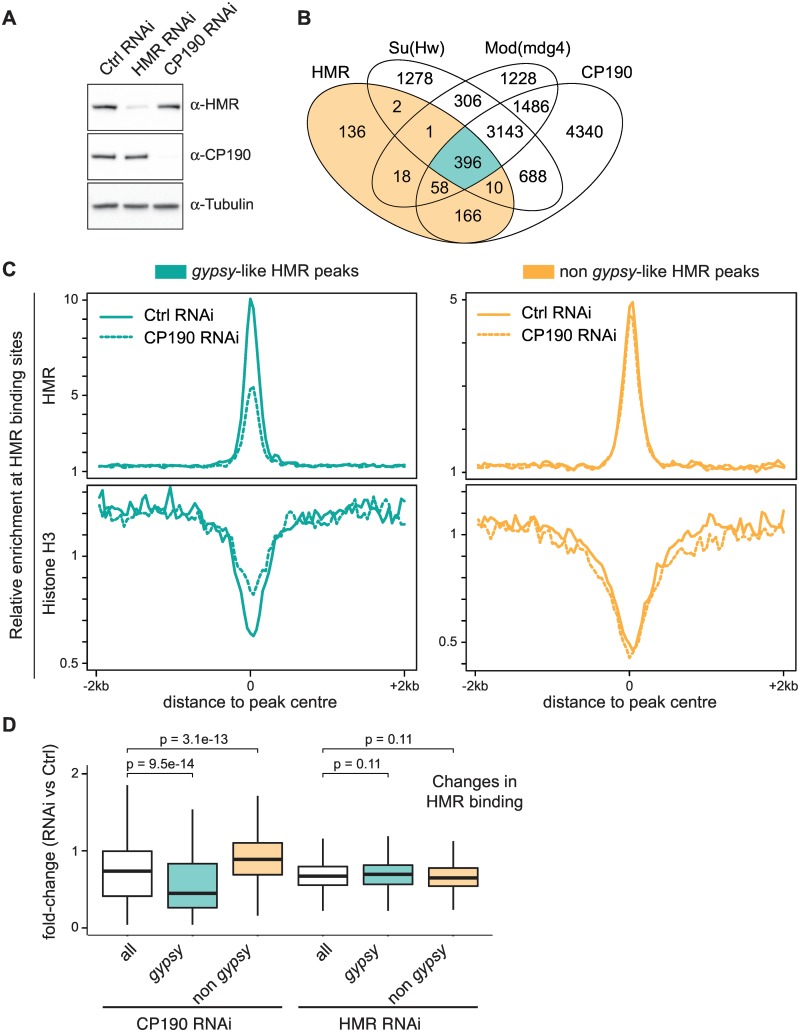
HMR genomic localization to *gypsy*-like insulator sites is dependent on CP190. **(A)** Western Blot of cell lysates after treatment with specific and control dsRNA shows an efficient knock-down of HMR and CP190. **(B)** Venn diagram of the overlap between HMR, CP190, Mod(mdg4) and Su(Hw) peaks [39] classifying HMR peaks as *gypsy*-like (highlighted in *green*) and non *gypsy*-like (highlighted in *orange*). **(C)** Composite analysis of HMR ChIP signals and Histone H3 ChIP signals at genomic HMR peak positions according to the groups defined in **(B)**. **(D)** Quantification of the fold-change of HMR ChIP enrichment upon CP190 RNAi and HMR RNAi. Box plots represent the fold-change of normalized HMR ChIP tag number aligned to 200 bp wide HMR peak regions. Peak regions with less than 50 aligned tags were excluded from the analysis. Significance of difference was estimated with p-values calculated with Wilcoxon rank sum test [72].

A key element for the formation of insulator complexes at *gypsy*-like elements is the presence of the CP190 adaptor protein. A reduction of CP190 levels has been shown to strongly affect binding of insulator proteins to these elements but not to others [43]. To test whether CP190 also impacts the binding of HMR to *gypsy*-like binding sites, we performed RNAi knock-down experiments to reduce CP190 protein level (Fig 4A) and measured HMR binding. Strikingly, we observe a substantial reduction of HMR binding only for the *gypsy*-like group of binding sites (Fig 4B and 4C), suggesting that HMR’s binding to the *gypsy*-like insulator class is indeed dependent on CP190. A HMR RNAi knock-down in contrast affects HMR binding equally in both classes (Fig 4D and S3 Fig). As insulator sites are known to contain less nucleosomes [51], nucleosome occupancy can serve as a proxy for insulator complex integrity at these sites [43]. We therefore performed a Histone H3 ChIP upon CP190 RNAi knock-down to monitor changes in insulator complex integrity [52]. Consistent with the importance of CP190 for maintaining the *gypsy* insulator, nucleosome occupancy only increases in the *gypsy*-like HMR binding sites (Fig 4C). Taken together, these results demonstrate an extensive colocalization of HMR with genomic insulator proteins, which play an important role in mediating its binding to chromatin.

### HMR borders HP1a domains at active promoters

**Fig 5:**
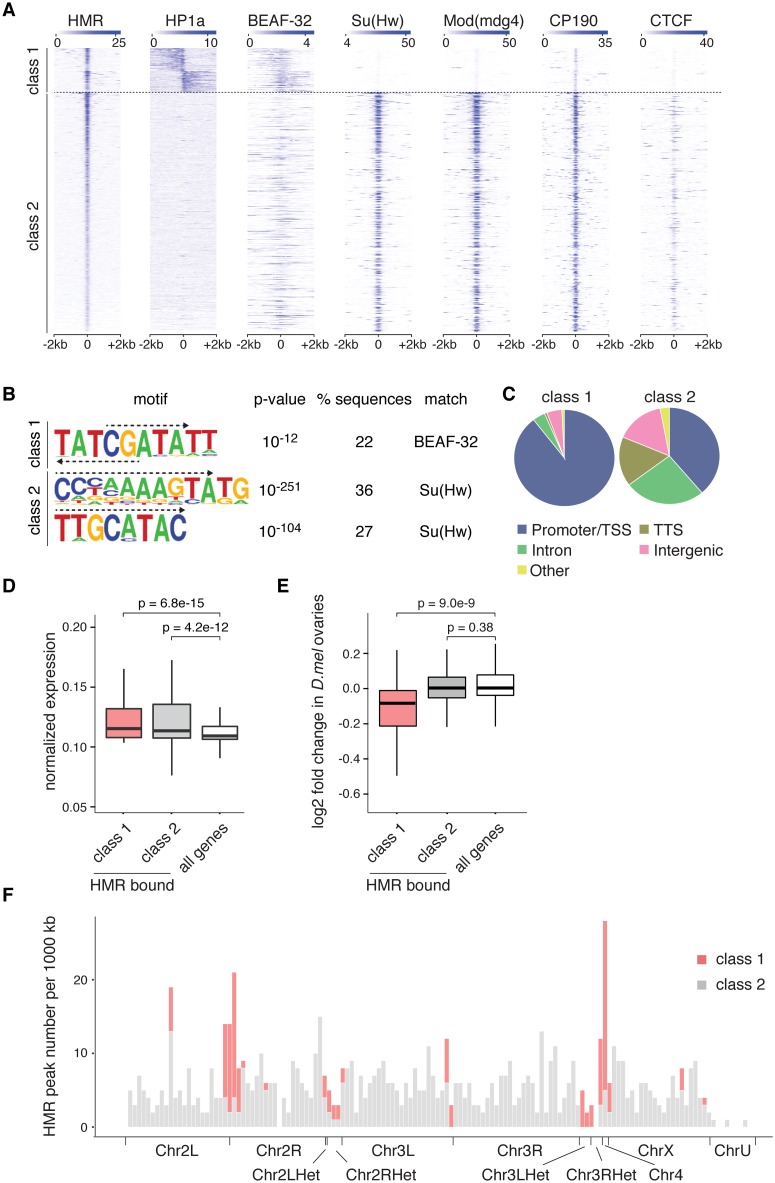
HMR borders HP1a together with BEAF-32 at the TSS of actively transcribed genes and enhances their transcription. **(A)** Heatmaps of HMR, HP1a, BEAF-32 [40], Su(Hw), Mod(mdg4), CP190 and CTCF [39] ChIP signals. All signals are centered around the HMR binding sites, clustered according to adjacent HP1a signals and sorted by HMR intensity. **(B)** Sequence motifs identified within HMR peak regions from class 1 and class 2 based on HOMER motif analysis. The corresponding motif logo, p-value of enrichment, percentage of regions with this motif, and putative binding factors are indicated. Dashed arrows mark the sequence that matches the published binding sites of Su(Hw) and BEAF-32 (see also S2A Fig). **(C)** Distribution of class 1 and class 2 HMR peaks among various genomic landmarks. **(D)** Box plot showing the normalized RNA expression of all genes and HMR-bound genes (promoter/TSS annotated) in class 1 and in class 2. S2 cells RNA expression levels were used according to [73]. Significance of difference was estimated with p-values calculated with Wilcoxon signed rank test [72]. **(E)** Box plot showing the log2 fold change of protein coding gene transcripts of all analyzed genes and HMR-bound genes (promoter/TSS annotated) in class 1 and in class 2 comparing *Hmr* mutant against wild type flies. The RNA-Seq data comes from experiments done in *D*. *melanogaster* ovaries [4]. Significance of difference was estimated with p-values calculated with Wilcoxon rank sum test [72]. For both box plots the box represents the interval that contains the central 50% of the data with the line indicating the median. The length of the whiskers is 1.5 times the interquartile distance (IQD). **(F)** Histogram showing HMR peak density across the annotated *D*. *melanogaster* genome. Class 1 HMR binding sites are enriched at region 31, centromere-proximal regions and the 4th chromosome.

Although a large portion of HMR binding sites is associated with *gyspy and gypsy-*like insulators, there is a considerable number of HMR-bound peaks that do not localize with Su(Hw), Mod(mdg4) and CP190 (Fig 4B). We noticed that many of these non *gypsy*-like sites are in close proximity to HP1a bound regions (Fig 1D). Indeed, when we sorted all HMR peaks according to the presence of HP1a in their proximity, we observed an almost complete lack of *gypsy* insulator binding proteins at these sites (Fig 5A). Consistent with the lack of Su(Hw) binding to this class of HMR peaks, a motif search revealed no enrichment of the Su(Hw) recognition site among those peaks but rather an enrichment for BEAF-32 binding sites (Fig 5B). To better understand a possible role of HMR at these sites, which we termed class 1 binding sites, we analyzed them with regards to their annotation. Interestingly, almost all HP1a-associated HMR binding sites (90%) are in close proximity to transcriptional start sites (TSS), whereas the other HMR binding sites show a somewhat broader distribution among various functional elements (Fig 5C). Strikingly, HMR binds very closely to the TSS at the boundary between HP1a containing domains and the gene body (S4A Fig). The genes in proximity of these HMR binding sites are classified as transcriptionally active suggesting that HMR might prevent the repressive influence of HP1a on neighbouring genes (Fig 5D). To investigate whether HMR loss has an impact on HP1a or H3K9me3 domains at these genomic regions, we performed HP1a ChIP and H3K9me3 ChIP upon HMR knockdown. However, we could not confirm extensive spreading of the heterochromatin marks HP1a or H3K9me3 after HMR loss (S4C and S4D Fig). Nevertheless, the genes associated with this class of HMR binding sites are transcriptionally down-regulated in *Hmr* mutant larvae and ovaries (Fig 5E, S4E Fig and [5]). This seems to be particularly important within regions that are rich in heterochromatin such as the 4th chromosome or the pericentromeric regions where we find the class of HP1a-associated binding sites highly enriched (Fig 5F). In summary, we can classify HMR’s genomic binding sites into two groups: One being associated with *gypsy* insulators, and another one associated with active promoters in pericentromeric heterochromatin where HMR borders HP1a-containing chromatin regions together with BEAF-32 and potentially promotes gene transcription.

## Discussion

HMR localizes to centromeric and pericentromeric regions in *D*. *melanogaster* cell lines as well as in mitotically dividing embryonic cells where it has been suggested to act as a repressor of transposable elements [3–5]. Mutations in *Hmr* lead to overexpression of satellite DNA and transposable elements in ovaries and larvae [4]. Such a derepression is also observed in hybrid flies [53], where HMR and LHR levels are higher than the ones in pure species and result in a widespread distribution of the HMR/LHR complex [3]. To better understand the targeting principles that mediate HMR binding within the *D*. *melanogaster* genome, we wondered whether we could identify HMR binding sites by applying ChIP-Seq in the *D*. *melanogaster* S2 cell line. Combining this approach with RNAi mediated knockdown experiments we uncover a strong colocalization of HMR with *gypsy* insulator binding sites and demonstrate that HMR binding to these sites depends on the presence of the residing insulator protein complex. Notably, HMR associates only with a subset of all Su(Hw) binding sites, but almost all those sites can be classified as *gypsy*-like sites bound by CP190 and mod(mdg4) in addition to Su(Hw).

Besides dispersed binding of HMR at genomic *gypsy* insulator sites along the chromosome arms, we observe dense clusters of HMR binding sites around the centromere and on the 4th chromosome where it potentially serves to separate HP1a binding domains from highly active genes. This dense clustering of binding sites around the centromere correlates well with the strong colocalization of HMR signals with the centromeric H3 variant CID in immunolocalization experiments [3]. Due to its biochemical interaction and partial colocalization with the heterochromatin protein HP1a in *Drosophila* embryos, HMR has been suggested to be a *bona-fide* heterochromatin component [3,4,6,54]. However, in contrast to HP1a, we detect very distinct HMR binding sites within the genome. When we find HMR close to an HP1a binding domain, it rather borders it than covering the whole domain. The sharp HMR binding signals and the fact that almost all euchromatic HMR binding sites contain putative insulator elements, suggest a role of HMR in separating chromatin domains. A distinct boundary that separates constitutive heterochromatin from the core centromere has also been postulated by Olszak and colleagues who suggest that transition zones between heterochromatin and euchromatin are hotspots for sites of CID misincorporation [55]. Unfortunately, centromeres are notoriously difficult to study by next generation sequencing due to their highly repetitive nature [56,57]. In addition, the microscopic resolution is not sufficiently high to allow a distinction between a binding to the core centromere chromatin and the chromatin immediately adjacent to it. Therefore, we cannot rule out the possibility that HMR binds large domains at the central region of the *Drosophila* centromere. However, the fact that the purification of chromatin containing the centromeric H3 variant CID did not identify HMR [58], suggests that it may very well also form a boundary between pericentromeric heterochromatin and the core centromere. To which extent and by which mechanism HMR fulfils a functional role at these genomic sites remains to be elucidated.

The genomic sites, where we find HMR bound next to an HP1a domain, are highly enriched for recognition sites of the insulator protein BEAF-32. Interestingly, a depletion of BEAF-32 in S2 cells results in an increased rate of mitotic defects [45], which is very reminiscent of the phenotype detected when HMR is depleted [3]. Similarly to flies carrying a mutation in the *Hmr* gene, flies in which BEAF-32 is only contributed maternally have defects in female fertility [59,60]. BEAF-32’s role in maintaining associated promoter regions in an environment that facilitates high transcription levels [61] has been suggested to be functionally relevant for this phenotype [45]. Strikingly, we find most HMR/BEAF-32 binding sites located between HP1a containing heterochromatin and the transcription start site of a highly active gene. HP1a chromatin might fulfil a repressive function at these genomic regions and HMR might block this repressive impact on the neighbouring gene body. However, we do not see extensive spreading of HP1a or H3K9me3 upon HMR knockdown suggesting that the repressive effect is not directly mediated by HP1a binding or the HMR knock down not efficient enough. As there is evidence that HP1a can also promote gene transcription [62], HMR may also function as a co-activator for HP1a. Currently, we therefore consider HMR binding next to HP1a containing chromatin as a unifying feature of transcriptionally affected genes but can only speculate about potential mechanism by which HMR exerts its function.

Although HMR depletion has a substantial effect on the transcription of multiple transposons, we find HMR only enriched at the 5' insulator region of the *gypsy* or *gtwin* retrotransposons and to similar sites within the genome that are presumably derived from these elements. These sites are occupied by insulator proteins Su(Hw), CP190 and Mod(mdg4) and often display enhancer blocking activity in transgenic assays [43,63–65]. Artificial targeting of HMR to DNA placed between an enhancer and a promoter of a reporter gene can block the transcription activity [3], suggesting that HMR may indeed play a role in setting up endogenous boundary elements. Similar to what is known for Su(Hw), HMR binding to this class of binding sites is dependent on the presence of the structural protein CP190, which has a key function in the stabilization of insulator protein complexes [22]. However, as we do not observe a strong physical interaction between CP190 and HMR, the loss of HMR binding upon a reduction of CP190 levels may also be the result of increased nucleosome occupancy. Such increase in Histone H3 binding cannot be observed upon HMR removal suggesting that HMR acts downstream of CP190. Interestingly, CP190 loss impairs HMR binding to *gypsy*-like insulator sites but has weak effect on HMR binding to sites containing BEAF-32 recognition motifs. Notably, in contrast to BEAF-32, CP190 is not required for oogenesis [66], suggesting that the lack of HMR binding to the class 1 sites may be responsible for the female sterility phenotype observed in *Hmr* mutant flies.

How can we integrate our findings with the lethal phenotype of increased HMR/LHR levels in male hybrids? It is tempting to speculate that multiple additional binding sites that are observed in hybrids and on polytene chromosomes of fly strains over-expressing HMR [3] constitute boundary regions. An increased binding to such boundaries, which have been shown to cluster and form aggregates *in vivo* [48,67,68], may trigger a massive change in nuclear architecture. In turn, this could indirectly activate multiple transposable elements similar to what is observed when centromere clustering is disturbed [69]. Such a disturbed nuclear architecture may then trigger the activation of a cell cycle checkpoint which has been previously suggested to be a major cause of hybrid lethality [70,71].

Altogether, our data provide a novel link between HMR and *cis*-regulatory elements bound by insulator proteins. We speculate that divergent evolution of such genomic elements and their corresponding binding factors in sibling species is triggering hybrid incompatibilities.

## Supporting information

S1 Fig Control experiments of HMR ChIP-Seq studies. **(A)** Venn diagram showing the lack of overlap between HMR peaks (peaks identified in at least two out of three independent biological replicates, highlighted in *grey*) and control IgG (anti-FLAG) ChIP peaks (pool of peaks from two independent biological replicates). **(B)** Changes in HMR ChIP enrichment upon HMR RNAi versus a control RNAi (GST) in two biological replicates. Each data point represents a mapped HMR peak. The scatter plot on the left displays fold changes of normalized HMR ChIP tag number mapped to a 200 bp HMR peak region in two biological replicates. Peak regions with less than 50 aligned tags were excluded from the analysis. The histogram on the right shows the frequency of peaks displaying a reduction of HMR binding upon knock-down. Shown are average values of replicate 1 and replicate 2. **(C)** ChIP-qPCR showing specific HMR enrichment at HMR binding sites. HMR ChIP is enriched for HMR binding sites in both wild type and HMR-Flag_2_ expressing cells. FLAG ChIP is enriched for HMR binding sites only in HMR-Flag_2_ expressing cells but not in wild type cells lacking the Flag_2_ epitope. Data are represented as mean ± SD of three technical replicates. **(D)** Genome browser view of HMR ChIP, HP1a ChIP and control IgG ChIP signal at a large centromere-proximal region at the right arm of the 2nd chromosome.
(TIF)

S2 Fig Overlap of HMR binding sites with known insulator regions and repetitive DNA. **(A)** Sequence motifs identified within Su(Hw) [39] and BEAF-32 [40] peak regions. The corresponding motif logo, p-value of enrichment and percentage of regions with this motif are indicated. Dashed arrows mark the sequence that matches the published binding sites of Su(Hw) and BEAF-32. (B) Genome browser view of ChIP signals showing combinatoric binding pattern for HMR and the insulator proteins CP190, Mod(mdg4), Su(Hw), CTCF [39] and BEAF-32 [40]. (C) Su(Hw) and HP1a ChIP tag enrichment at repetitive DNA elements. Each point in the scatter plot represents the enrichment (log2 fold) over input and the RPKM of an individual repeat from Repbase. Repeats with less than 2-fold enrichment are not displayed. (D) ChIP tag density of HMR and the *gypsy*-insulator proteins CP190, Mod(mdg4), Su(Hw) [39] across the *gypsy-twin* repeat.
(TIF)

S3 Fig Selective effect of CP190 RNAi on HMR binding to *gypsy*-like elements. Composite analysis of HMR ChIP signal and Histone H3 ChIP signal at genomic HMR peak positions according to the groups defined in Fig 4B. The ChIP signals were obtained upon control RNAi and HMR RNAi. The HMR ChIP signals are similarly affected in both groups, whereas Histone H3 ChIP signals are retained.
(TIF)

S4 Fig Additional information for HP1a-associated HMR binding sites. **(A)** Composite analysis of HMR, HP1a and BEAF-32 ChIP signals at class 1 genomic sites relative to the transcriptional start site (TSS) and the gene body. Shown are normalised and scaled read density plots **(B)** Peak overlap of HMR with peaks of the insulator proteins CP190, Mod(mdg4), Su(Hw), CTCF [39] and BEAF-32 [40] for class 1 and for class 2 HMR binding sites. **(C)** Composite analysis of HP1a and H3K9me3 ChIP signals at class 1 HMR binding sites after HMR knockdown. Class 1 is defined in Fig 5A but oriented according to HP1a ChIP signal. **(D)** Western Blot analysis on cell lysates to assay protein levels after HMR knockdown. Tubulin protein detection served as control. **(E)** Same as described in Fig 5E, but the RNA-Seq data comes from experiments done in *D*. *melanogaster* male larvae [4].
(TIF)

S1 Table List of primers used for CRISPR/*cas9* genome editing, RNAi experiments and ChIP Real-Time PCR. List of primers used in this study. Primers used in ChIP Real-Time PCR were designed with help of Primer3.
(DOCX)

S2 Table ChIP-Seq sample overview and number of uniquely aligned sequence reads. ChIP-Seq sample overview and number of uniquely aligned sequence reads. The percentage of uniquely mapped reads in ChIP-Seq experiments can largely vary and depends on the nature of the ChIPed protein. Proteins that bind repetitive regions (such as HMR or HP1a) give substantially lower percentages of uniquely mapped reads.
(DOCX)

S3 Table HMR peaks used for downstream analysis. HMR peak list derived from HOMER peak calling on three biological replicates (Fig 1A). First three columns provide information on the peak position within the genome (chromosome, peak start and end using dm3), followed by peak annotation obtained from ChIPseeks implementation of HOMER (Fig 5C) and classification according to adjacent HP1a signals (Fig 5A).
(XLSX)

S1 Methods Supporting information on methods. *Hmr* gene editing using CRISPR/*cas9*, extended ChIP Real-Time PCR methods, extended ChIP-seq data analysis methods.
(DOCX)

## Abbreviations

BEAF-32: Boundary element associated factor 32
ChIP: Chromatin immunoprecipitation
CID: Centromer identifier
CP190: Centrosomal Protein 190
CRISPR: clustered, regularly interspaced, short palindromic repeats
CTCF: CCCTC binding factor
DGRC: *Drosophila* genomics resource center
gRNA: guide RNA
GST: Glutathione S-transferase
*gtwin*: *gypsy-twin*
HMR: Hybrid male rescue
HP1a: Heterochromatin Protein 1a
LHR: Lethal hybrid rescue
Mod(mdg4): Modifier of mdg4
RNAi: RNA interference
Su(Hw): Suppressor of Hairy wing
TSS: Transcription start site
