# Supplementary figures and images for: The Drosophila speciation factor HMR localizes to genomic insulator sites
Source: PLoS One. 2017 Feb 16;12(2):e0171798. doi: 10.1371/journal.pone.0171798 (PMC5312933; doi:10.1371/journal.pone.0171798)

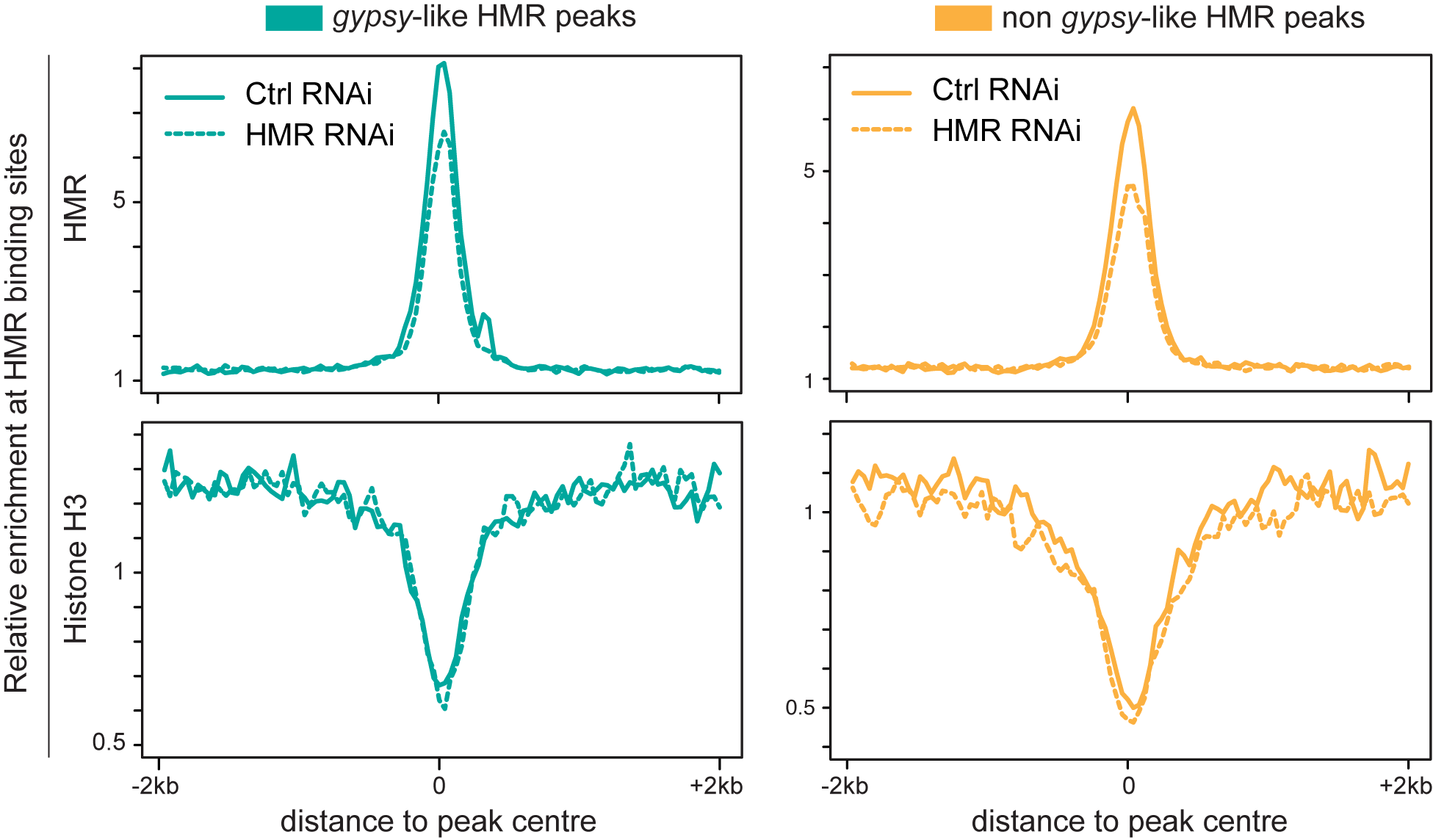

Supplement: S3 Fig — Composite analysis of HMR ChIP signal and Histone H3 ChIP signal at genomic HMR peak positions according to the groups defined in Fig 4B. The ChIP signals were obtained upon control RNAi and HMR RNAi. The HMR ChIP signals are similarly affected in both groups, whereas Histone H3 ChIP signals are retained. (TIF) [file pone.0171798.s003.tif]

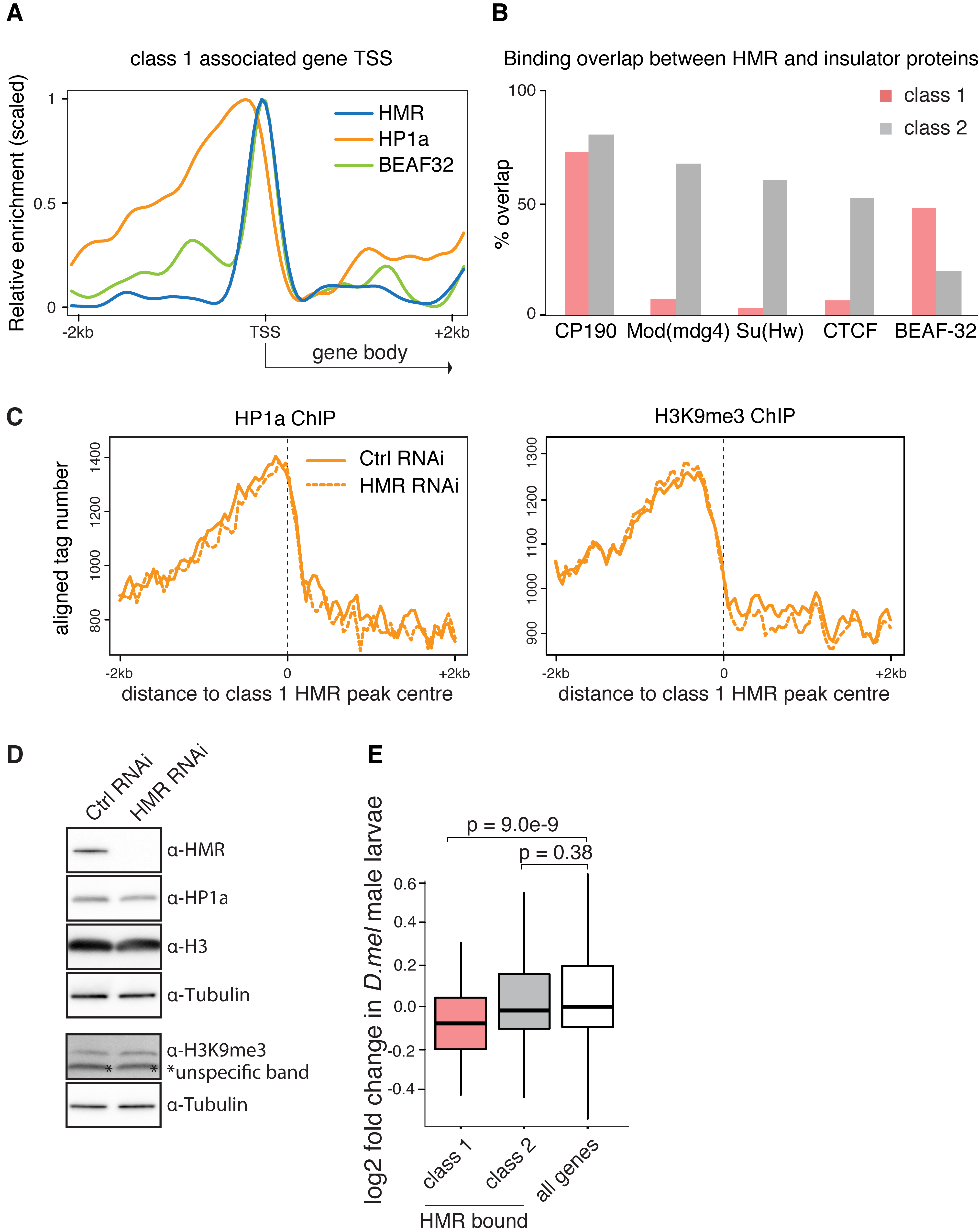

Supplement: S4 Fig — (A) Composite analysis of HMR, HP1a and BEAF-32 ChIP signals at class 1 genomic sites relative to the transcriptional start site (TSS) and the gene body. Shown are normalised and scaled read density plots (B) Peak overlap of HMR with peaks of the insulator proteins CP190, Mod(mdg4), Su(Hw), CTCF [39] and BEAF-32 [40] for class 1 and for class 2 HMR binding sites. (C) Composite analysis of HP1a and H3K9me3 ChIP signals at class 1 HMR binding sites after HMR knockdown. Class 1 is defined in Fig 5A but oriented according to HP1a ChIP signal. (D) Western Blot analysis on cell lysates to assay protein levels after HMR knockdown. Tubulin protein detection served as control. (E) Same as described in Fig 5E, but the RNA-Seq data comes from experiments done in D. melanogaster male larvae [4]. (TIF) [file pone.0171798.s004.tif]
