# Supplementary material for: The Drosophila speciation factor HMR localizes to genomic insulator sites
Source: PLoS One. 2017 Feb 16;12(2):e0171798. doi: 10.1371/journal.pone.0171798 (PMC5312933; doi:10.1371/journal.pone.0171798)
Supplement: S1 Table — List of primers used in this study. Primers used in ChIP Real-Time PCR were designed with help of Primer3. (DOCX) [file pone.0171798.s005.docx]

| **Name** | **Sequence (5’-3’)** | **Publication** |
| --- | --- | --- |
| ***CRISPR/cas9 system based HMR epitope tagging*** | | |
| Lig4 RNAi f | TAATACGACTCACTATAGGGCCCAATGATCCAAAGTGTTTTTGCA | [1] |
| Lig4 RNAi r | TAATACGACTCACTATAGGGAAGTAGGATGCCTTCGCGA | [1] |
| oligo scaffold | GTTTTAGAGCTAGAAATAGCAAGTTAAAATAAGGCTAGTCCGTTATCAACTTGAAAAAGTGGCACCGAGTCGGTGC | [1] |
| oligo CRISPR target with T7 prom. | TAATACGACTCACTATAGCCACCGCCTTAGCTCTCGAAACTTTGTTTTAGAGCTA | this study |
| primer antis. scaffold | GCACCGACTCGGTGCCACT | [1] |
| U6-gRNA sense | GCTCACCTGTGATTGCTCCTAC | [1] |
| U6-gRNA antisense | GCTTATTCTCAAAAAAGCACCGACTCGGTGCCACT | [1] |
| HMRtar sense | TGGGCCTACGCCGTCGGTAACTTGTCCACGGCCAGTCAGGATACACTGCTCGGCAAGATGACGCAGCTGTTCTCTAAATACGCCAAGGTCAATCCGCCACCGCCTGGATCTTCCGGATGGCTCGAG | this study |
| HMRtar antisense | ACGGCGAAAGTTCTTACAGAGAATATGTATGACTAAACTACGTGTGCCAAAAGTTTCGAGAGGAAGTTCCTATTCTCTAGAAAGTATAGGAACTTCCATATG | this study |
| *Hmr* CDS sense | TATAAGCAGGTGAAGCCGAAC | this study |
| *Hmr* downstream antisense | TGCCCTCATCGCTATCATTCTG | this study |
| ***RNAi knockdown experiments*** | | |
| CP190 RNAi f | TAATACGACTCACTATAGGGCCTGGCTGTGCCTGAGA | [2] |
| CP190 RNAi r | TAATACGACTCACTATAGGGCTGGTAGACTTATGTCCGAAA | [2] |
| GST RNAi f | TTAATACGACTCACTATAGGGAGAAGTTTGAATTGGGTTTGGAGTTTCC | [3] |
| GST RNAi r | TTAATACGACTCACTATAGGGAGATCGCCACCACCAAACGTGG | [3] |
| HMR RNAi f | TTAATACGACTCACTATAGGGAGAGATGTGGAGGTCATAGAGAATCCGCCAATG | [3] |
| HMR RNAi r | TTAATACGACTCACTATAGGGAGAACCTTGTTGTGCAGGGAGTCCTCCGTC | [3] |
| ***ChIP Real-Time PCR*** | | |
| 2L:302129-302248 for | CACAGCAACGAAGCTCTCTG | this study |
| 2L:302129-302248 rev | AGCATAGTGACCCGCATCTC | this study |
| *3R:23793216-23793267 for* | GAGCAAGAACAGCAGCTACTTTGT | this study |
| *3R:23793216-23793267 rev* | CACCTTGACGTTGTTGGGAAT | this study |
| *3RHet:2107224-2107336 for* | AACCCTATCCAAATTTCGAACC | this study |
| *3RHet:2107224-2107336 rev* | AGCCAAGATGAAGTCGATGC | this study |
| *4:855631-855744 for* | TAAACTCAGCCCTGCATTCC | this study |
| *4:855631-855744 rev* | GTGTTAAACCAATCCGAGACATC | this study |
| *2RHet:369982-370075 for* | CATTTGACTTCTTCGACACGAC | this study |
| *2RHet:369982-370075 rev* | GACACTGATTTACACAAAGCACAAC | this study |
| *2RHet:370407-370487 for* | TGCATACCCTACAAATAGTTTTGC | this study |
| *2RHet:370407-370487 rev* | TTGATCGGCTAAGTGAAGTGG | this study |

**S1 Table.** List of primers used in this study. Primers used in ChIP Real-Time PCR were designed with help of Primer3 [4].

**References**

1. Böttcher R, Hollmann M, Merk K, Nitschko V, Obermaier C, Philippou-Massier J, et al. Efficient chromosomal gene modification with CRISPR/cas9 and PCR-based homologous recombination donors in cultured Drosophila cells. Nucleic Acids Res. Oxford University Press; 2014;42: e89–e89. doi:10.1093/nar/gku289
2. Van Bortle K, Ramos E, Takenaka N, Yang J, Wahi JE, Corces VG. Drosophila CTCF tandemly aligns with other insulator proteins at the borders of H3K27me3 domains. Genome Res. 2012;22: 2176–2187. doi:10.1101/gr.136788.111
3. Thomae AW, Schade GOM, Padeken J, Borath M, Vetter I, Kremmer E, et al. A Pair of Centromeric Proteins Mediates Reproductive Isolation in Drosophila Species. Dev Cell. 2013;27: 412–424. doi:10.1016/j.devcel.2013.10.001
4. Koressaar T, Remm M. Enhancements and modifications of primer design program Primer3. Bioinformatics. Oxford University Press; 2007;23: 1289–1291. doi:10.1093/bioinformatics/btm091
