# Supplementary material for: The Drosophila speciation factor HMR localizes to genomic insulator sites
Source: PLoS One. 2017 Feb 16;12(2):e0171798. doi: 10.1371/journal.pone.0171798 (PMC5312933; doi:10.1371/journal.pone.0171798)
Supplement: S2 Table — ChIP-Seq sample overview and number of uniquely aligned sequence reads. The percentage of uniquely mapped reads in ChIP-Seq experiments can largely vary and depends on the nature of the ChIPed protein. Proteins that bind repetitive regions (such as HMR or HP1a) give substantially lower percentages of uniquely mapped reads. (DOCX) [file pone.0171798.s006.docx]

| **Treatment** | **Sample** | **Number of reads** | **Number of unique reads** |
| --- | --- | --- | --- |
| untreated | HMR_1 | 20989662 | 7321851 |
| untreated | HP1a_1 | 21130231 | 9615590 |
| untreated | IgG_1 | 15461190 | 8941371 |
| untreated | Input_1 | 17888908 | 10427301 |
| untreated | HMR_2 | 22695222 | 11790768 |
| untreated | HP1a_2 | 21119605 | 3793451 |
| untreated | IgG_2 | 29198102 | 17068323 |
| untreated | Input_2 | 23224706 | 14681351 |
| untreated | HMR_3 | 21035775 | 11727510 |
| untreated | Input_3 | 23103165 | 14734701 |
| untreated | HP1a_4 | 27968435 | 13525876 |
| untreated | Input_4 | 34527460 | 21798351 |
| Ctrl RNAi | HMR_CtrlRNAi_1 | 24593353 | 8637377 |
| Ctrl RNAi | HP1a_CtrlRNAi_1 | 7603697 | 765717 |
| Ctrl RNAi | H3_CtrlRNAi_1 | 21646028 | 13497422 |
| Ctrl RNAi | H3K9me3_CtrlRNAi_1 | 20709056 | 9610889 |
| Ctrl RNAi | Input_CtrlRNAi_1 | 23510417 | 14443029 |
| HMR RNAi | HMR_HMRRNAi_1 | 22969101 | 11868648 |
| HMR RNAi | HP1a_HMRRNAi_1 | 18370927 | 7419588 |
| HMR RNAi | H3_HMRRNAi_1 | 16887197 | 10562127 |
| HMR RNAi | H3K9me3_HMRRNAi_1 | 19310617 | 8553421 |
| HMR RNAi | Input_HMRRNAi_1 | 22252385 | 14445163 |
| CP190 RNAi | HMR_CP190RNAi_1 | 23814936 | 10825131 |
| CP190 RNAi | H3_CP190RNAi_1 | 20909517 | 13085885 |
| CP190 RNAi | Input_CP190RNAi_1 | 24311469 | 15526600 |
| Ctrl RNAi | HMR_CtrlRNAi_2 | 24910232 | 14947403 |
| Ctrl RNAi | Input_CtrlRNAi_2 | 19192206 | 11772305 |
| HMR RNAi | HMR_HMRRNAi_2 | 25880670 | 15528028 |
| HMR RNAi | Input_HMRRNAi_2 | 26225419 | 16101359 |

**S2 Table.** ChIP-Seq sample overview and number of uniquely aligned sequence reads. The percentage of uniquely mapped reads in ChIP-Seq experiments can largely vary and depends on the nature of the ChIPed protein. Proteins that bind repetitive regions (such as HMR or HP1) give substantially lower percentages of uniquely mapped reads [1,2].

**References**

1. Jung YL, Luquette LJ, Ho JWK, Ferrari F, Tolstorukov M, Minoda A, et al. Impact of sequencing depth in ChIP-seq experiments. Nucleic Acids Res. 2014;42: e74–e74. doi:10.1093/nar/gku178
2. Bailey T, Krajewski P, Ladunga I, Lefebvre C, Li Q, Liu T, et al. Practical Guidelines for the Comprehensive Analysis of ChIP-seq Data. Lewitter F, editor. PLoS Comput Biol. 2013;9: e1003326–8. doi:10.1371/journal.pcbi.1003326
