# Supplementary material for: The Drosophila speciation factor HMR localizes to genomic insulator sites
Source: PLoS One. 2017 Feb 16;12(2):e0171798. doi: 10.1371/journal.pone.0171798 (PMC5312933; doi:10.1371/journal.pone.0171798)
Supplement: S1 Methods — Hmr gene editing using CRISPR/cas9, extended ChIP Real-Time PCR methods, extended ChIP-seq data analysis methods. (DOCX) [file pone.0171798.s008.docx]

**Supplemental methods**

***Hmr* gene editing using CRISPR/*cas9***

Endogenous tagging of *Hmr* in S2 cells was performed with support of Prof. Klaus Förstemann and colleagues and was performed exactly as described in [1]. We used *D. melanogaster* S2-DRSC cells in combination with U6-driven guide-RNA construct generated by overlap extension PCR. *Hmr*-specific reagents are listed in S1 Table.

**Extended ChIP Real-Time PCR methods**

Specific primers (S1 Table) were designed with help of Primer3 [2]. After purification, input DNA was diluted 500-fold, immunoprecipitated DNA was diluted 10-fold before Real-Time PCR. Real-Time PCR was performed in 10 μL reaction volume with 5 μL 2x Fast SYBR Green master mix (Applied Biosystems), 1 µL 3 mM Primer forward, 1 µL 3 mM Primer reverse, 2 µL DNA template and 1 µL H_2_O on a LightCycler 480 II (Roche). The PCR program was 20 seconds at 95°C; 45 cycles of 95°C for 3 seconds and 60°C for 30 seconds.

The sample’s Ct values (number of cycles required for the fluorescent signal to cross the threshold) reported by the LightCycler 480 II (Roche) software were used to calculate the percentage of immunoprecipitated DNA with respect to the input DNA. The percentage (Input %) value is

% input = e ^ΔCt^  x df x vf x 100

with ΔCt = Ct (Input) - Ct (ChIP), e = primer pair efficiency (close to 2 or equals 2) calculated with LightCycler 480 II (Roche) software on a serial dilution of template DNA, df = Dilution factor taking dilution of DNA template into account (df = 10/500, see above) and vf = Volume factor taking starting volume of ChIP and Input into account [vf = starting volume(Input)/starting volume(ChIP)].

**Extended ChIP-seq data analysis methods**

The raw reads were aligned to the *D. melanogaster* genome assembly (UCSC dm3) using Bowtie (version 2.2.6) [3] and excluding chromosome Uextra [3]. Only uniquely mapped reads are kept using samtools (version 1.2) [4]. The raw read quality was accessed using FASTQC (version 11.5) [5] and reads filtering was performed using FastX (version 0.0.13) [6]. Sequencing tracks of both fold enrichment and log (of base 2) transformation with parameter settings *–m FE* and *–m logLR –p 0.00001* were generated using MACS (version 2.1.1) [7], which were then visualized using IGB [8] and IGV [9] genome viewers. Peak calling was performed using HOMER 4.8 with parameter settings *-style factor -size 200 -fragLength 200 -inputFragLength 200* [10]. Motif search and peak annotation were performed using ChIPseeks implementation of HOMER [11].

For downstream analysis, peaks identified in two out of three biological replicates were taken. Downstream analysis steps were performed using Python and R and parts of data preprocessing was done using ChipPeakAnno [12]. For the clustering of HMR peaks according to adjacent HP1a ChIP signals, three clusters were generated with K-means algorithm [13].

For repeat analysis, reads from ChIP-Seq experiments were mapped to RepBase version 19.10 [14] using bowtie [3]. Only unique reads were kept for analysis. For each repetitive element log (of base 2) fold change was calculated. For the read density tracks, deepTools (version 2.3.3.5) [15] with parameter sets *--ratio ratio --pseudocount=1* was utilized to normalize against the control.

Following genome-wide binding data sets derived from S2 cells (unless stated otherwise) were used: CP190, Su(Hw), CTCF and mod(mdg4) from GEO GSE41354 [16], BEAF-32 from GEO GSE32815 [17]. RNA expression data for untreated S2 cells was taken from GEO GSE46020. For *D. melanogaster* larvae and ovaries, RNA-Seq data were taken from NCBI BioProject PRJNA236022 [18] and analyses were performed with cuffdiff 2 [19].

**References**

1. Böttcher R, Hollmann M, Merk K, Nitschko V, Obermaier C, Philippou-Massier J, et al. Efficient chromosomal gene modification with CRISPR/cas9 and PCR-based homologous recombination donors in cultured Drosophila cells. Nucleic Acids Res. Oxford University Press; 2014;42: e89–e89. doi:10.1093/nar/gku289
2. Koressaar T, Remm M. Enhancements and modifications of primer design program Primer3. Bioinformatics. Oxford University Press; 2007;23: 1289–1291. doi:10.1093/bioinformatics/btm091
3. Langmead B, Trapnell C, Pop M, Salzberg SL. Ultrafast and memory-efficient alignment of short DNA sequences to the human genome. Genome Biol. 2009;10: R25. doi:10.1186/gb-2009-10-3-r25
4. Li H, Handsaker B, Wysoker A, Fennell T, Ruan J, Homer N, et al. The Sequence Alignment/Map format and SAMtools. Bioinformatics. Oxford University Press; 2009;25: 2078–2079. doi:10.1093/bioinformatics/btp352
5. Andrews S. FATSQC, a quality control tool for high throughput sequence data. In: http://www.bioinformatics.babraham.ac.ukprojectsfastqc. 2010.
6. Hannon GJ. FASTX Toolkits FASTA/Q short reads preprocessing kit [Internet].
7. Zhang Y, Liu T, Meyer CA, Eeckhoute J, Johnson DS, Bernstein BE, et al. Model-based analysis of ChIP-Seq (MACS). Genome Biol. BioMed Central; 2008;9: R137. doi:10.1186/gb-2008-9-9-r137
8. Nicol JW, Helt GA, Blanchard SG, Raja A, Loraine AE. The Integrated Genome Browser: free software for distribution and exploration of genome-scale datasets. Bioinformatics. 2009;25: 2730–2731. doi:10.1093/bioinformatics/btp472
9. Thorvaldsdóttir H, Robinson JT, Mesirov JP. Integrative Genomics Viewer (IGV): high-performance genomics data visualization and exploration. Brief Bioinformatics. 2013;14: 178–192. doi:10.1093/bib/bbs017
10. Heinz S, Benner C, Spann N, Bertolino E, Lin YC, Laslo P, et al. Simple combinations of lineage-determining transcription factors prime cis-regulatory elements required for macrophage and B cell identities. Mol Cell. 2010;38: 576–589. doi:10.1016/j.molcel.2010.05.004
11. Chen T-W, Li H-P, Lee C-C, Gan R-C, Huang P-J, Wu TH, et al. ChIPseek, a web-based analysis tool for ChIP data. BMC Genomics. BioMed Central; 2014;15: 539. doi:10.1186/1471-2164-15-539
12. Zhu LJ, Gazin C, Lawson ND, Pagès H, Lin SM, Lapointe DS, et al. ChIPpeakAnno: a Bioconductor package to annotate ChIP-seq and ChIP-chip data. BMC Bioinformatics. 2010;11: 237. doi:10.1186/1471-2105-11-237
13. MacQueen J. Proceedings of the fifth Berkeley symposium on mathematical statistics and probability, volume 1: Statistics. Some methods for classification and …; 1967.
14. Bao W, Kojima KK, Kohany O. Repbase Update, a database of repetitive elements in eukaryotic genomes. Mobile DNA. 2015;6: 11. doi:10.1186/s13100-015-0041-9
15. Ramírez F, Ryan DP, Grüning B, Bhardwaj V, Kilpert F, Richter AS, et al. deepTools2: a next generation web server for deep-sequencing data analysis. Nucleic Acids Res. Oxford University Press; 2016;44: W160–5. doi:10.1093/nar/gkw257
16. Ong C-T, Van Bortle K, Ramos E, Corces VG. Poly(ADP-ribosyl)ation Regulates Insulator Function and IntrachromosomalInteractions in Drosophila. Cell. Elsevier Inc; 2013;155: 148–159. doi:10.1016/j.cell.2013.08.052
17. Riddle NC, Minoda A, Kharchenko PV, Alekseyenko AA, Schwartz YB, Tolstorukov MY, et al. Plasticity in patterns of histone modifications and chromosomal proteins in Drosophila heterochromatin. Genome Res. 2011;21: 147–163. doi:10.1101/gr.110098.110
18. Satyaki PRV, Cuykendall TN, Wei KH-C, Brideau NJ, Kwak H, Aruna S, et al. The hmr and lhr hybrid incompatibility genes suppress a broad range of heterochromatic repeats. Malik HS, editor. PLoS Genet. Public Library of Science; 2014;10: e1004240. doi:10.1371/journal.pgen.1004240
19. Trapnell C, Hendrickson DG, Sauvageau M, Goff L, Rinn JL, Pachter L. Differential analysis of gene regulation at transcript resolution with RNA-seq. Nat Biotechnol. 2013;31: 46–53. doi:10.1038/nbt.2450
